# Mapping the Genomic Limits of De-Extinction in the Face of Ancient DNA Degradation

**DOI:** 10.1093/gbe/evaf251

**Published:** 2026-01-03

**Authors:** Jianqing Lin, Xinrui Long, Yan Gao, Wenhua Liu, M Thomas P Gilbert

**Affiliations:** Guangdong Provincial Key Laboratory of Marine Disaster Prediction and Prevention, Guangdong Provincial Key Laboratory of Marine Biotechnology, Institute of Marine Science, College of Science, Shantou University, Shantou, Guangdong 515063, China; International Joint Research Center for Marine Ecological Protection and Disaster Prevention, Shantou University, Shantou 515063, China; Guangdong Provincial Key Laboratory of Marine Disaster Prediction and Prevention, Guangdong Provincial Key Laboratory of Marine Biotechnology, Institute of Marine Science, College of Science, Shantou University, Shantou, Guangdong 515063, China; International Joint Research Center for Marine Ecological Protection and Disaster Prevention, Shantou University, Shantou 515063, China; Guangdong Provincial Key Laboratory of Marine Disaster Prediction and Prevention, Guangdong Provincial Key Laboratory of Marine Biotechnology, Institute of Marine Science, College of Science, Shantou University, Shantou, Guangdong 515063, China; International Joint Research Center for Marine Ecological Protection and Disaster Prevention, Shantou University, Shantou 515063, China; Department of Anthropology and Human Genetics, School of Life Sciences, Fudan University, Shanghai 200433, China; Guangdong Provincial Key Laboratory of Marine Disaster Prediction and Prevention, Guangdong Provincial Key Laboratory of Marine Biotechnology, Institute of Marine Science, College of Science, Shantou University, Shantou, Guangdong 515063, China; International Joint Research Center for Marine Ecological Protection and Disaster Prevention, Shantou University, Shantou 515063, China; Center for Evolutionary Hologenomics, The GLOBE Institute, University of Copenhagen, Copenhagen 1353, Denmark; University Museum, Norwegian University of Science and Technology, Trondheim 7012, Norway

**Keywords:** de-extinction, ancient DNA, sequencing depth, coverage

## Abstract

The de-extinction of species using genome-editing approaches depends on acquiring high-quality genomic information from the extinct target. However, the degraded nature of the ancient DNA (aDNA) that is typical for most extinct species, poses significant challenges to achieving comprehensive genome reconstruction. A systematic evaluation of the minimum sequencing effort that is required to reliably map the genome under varying DNA quality conditions to different reference genome remains lacking across different extinct species. Here, we systematically assess the impact of sequencing depth on genome coverage, heterozygosity estimation, and variant calling accuracy, when mapping both true aDNA data generated from the extinct Christmas Island rat (*Rattus macleari*), as well as in silico simulated modern- and ancient-like data generated from a modern relation (the brown rat, *Rattus norvegicus*), to the black rat (*Rattus rattus*) reference genomes. Our results demonstrate that even sequencing depths of 100× fail to yield stable heterozygosity estimates, and leave approximately 3.38% to 4.03% of its genome uncovered. These uncovered regions contained functionally relevant SNPs and indels, highlighting the limitations of reconstructing extinct genomes using reference sequences from extant relatives. Furthermore, simulations using computationally generated “degraded haploid and diploid” data based on the high-quality brown rat genome, revealed that false-positive SNPs primarily arise from insufficient coverage and low data quality, rather than aDNA damage (e.g. miscoding lesions, size of fragments, etc.) per se. These findings underscore the need to tailor sequencing depth standards by considering sample type, degradation level, and sequencing error profiles. This study provides a theoretical framework and methodological support for optimizing data strategies in aDNA research, and ultimately informing de-extinction efforts.

Significance Genome-editing-based de-extinction strategies critically rely on reconstructing extinct genomes using sequencing data, but there is a lack of systematic understanding about how much sequencing is sufficient across different levels of DNA degradation and varying reference genomes. This study demonstrates that even high sequencing depths (≥100×) leaves significant portions of the extinct genome uncovered (including functionally important variants), and yields unstable heterozygosity estimates. Furthermore, simulations reveal that false-positive SNPs arise primarily from low sequencing coverage and data quality, not aDNA damage itself. This work establishes that sequencing needs must be customized based on degradation and error profiles, offering a concrete framework to significantly improve the accuracy of future de-extinction projects.

## Introduction

Species extinction—the permanent loss of a biological lineage—is a fundamental concern in ecology (Raup 1986). In response, de-extinction has emerged as a field aiming at reversing such losses, primarily through three approaches: back-breeding (Richmond et al. 2016; Shapiro and Seddon 2017), somatic cell nuclear transfer (Iqbal et al. 2021), and genome editing of closely related extant species. Among these, genome editing offers the greatest potential for the majority of extinct species, particularly with advances in synthetic biology and molecular engineering (Takahashi and Yamanaka 2006; Takahashi et al. 2007; Chen et al. 2025).

Given the constraints of current technologies, it is unrealistic to expect that gene-editing could be used to create a lifeform with a genome sequence that is 100% identical to that of a now extinct form. Hence current attempts at de-extinction either plan to, or have already restored, traits that are perceived to resemble those in extinct species, by editing a limited number of genes. This is consistent with IUCN's definition of de-extinction (IUCN Species Survival Commission 2016). As such, the recovery and subsequent editing of 100% of the genomic information from the extinct species is not viewed as key to de-extinction, and so genome mapping gaps and false positive SNPs are acceptable. However, as our previous study (Lin et al 2022) demonstrated, mapping gaps and false positive SNPs are randomly and widely distributed throughout the genome. Therefore, a prior requisite of these approaches is the sequencing of sufficient genomic ancient DNA (aDNA) from the extinct organism, to provide accurately reconstructed sequences from genes related to key traits. Recent improvements in aDNA extraction and sequencing library preparation have enabled partial genome reconstructions for species including the woolly mammoth (Palkopoulou et al. 2015), passenger pigeon (Murray et al. 2017), thylacine (Feigin et al. 2018), aurochs (Park et al. 2015), and the Christmas Island rat (Lin et al. 2022). Yet the feasibility of genome recovery is constrained by environmental conditions that affect DNA preservation—cold, dry climates favor longevity, while warm or humid environments accelerate degradation (Hofreiter et al. 2001). Consequently, genome-scale data recovery remains restricted to relatively recent extinctions.

While gene editing and next-generation sequencing (NGS) technologies have been improved substantially, a critical challenge remains: How do aDNA quality and sequencing depth impact the accuracy and completeness of reconstructed genomes? Analyses using modern datasets have, for example, revealed how sequencing depth (the average number of times a single base is sequenced) and coverage (the percentage of the genome or target region actually sequenced) affect variant detection accuracy. For instance, mapping of human sequencing data to human genomes reveals that medium-depth (10–30×) can minimize the false positives (Xu et al. 2020), and a depth of 22.5× has been shown to reliably capture high-quality single nucleotide variants. However standardized benchmarks for working with ancient DNA from extinct species—especially under varied sample preservation conditions—are lacking.

To address this, the present study analyzes both real aDNA sequence data from the extinct Christmas Island rat (*Rattus macleari)* and simulated data derived from its modern relative, the brown rat (*Rattus norvegicus*).Firstly, we simulated modern- and ancient-like haploid and diploid short-read data respectively, from the brown rat (*R. norvegicus*). We than mapped these back to the reference genome of the brown rat, to investigate how real SNPs are detected as sequencing depth increases in the absence of species evolutionary divergence. Then, we mapped these four sets of simulated data, as well as the real aDNA sequence from the extinct Christmas Island rat (*R. macleari*), to their modern relative, the black rat (*Rattus rattus*). This was done to simulate the process of genome reconstruction in de-extinction and ancient DNA research, and to explore the sources of false positive SNPs, in the context of evolutionary divergence. We aimed to systematically assess the impact of sequencing depth on genome coverage, heterozygosity estimation, and identify the minimal genomic sequencing data necessary to identify genomic variation exiting between the extinct and extant species. In doing so we aimed to provide a foundation that would enable similar analyses to be performed in the context of both ancient genome reconstruction, and potentially species de-extinction attempts.

## Results

### Same-Species Mapping

**Fig. 1.**
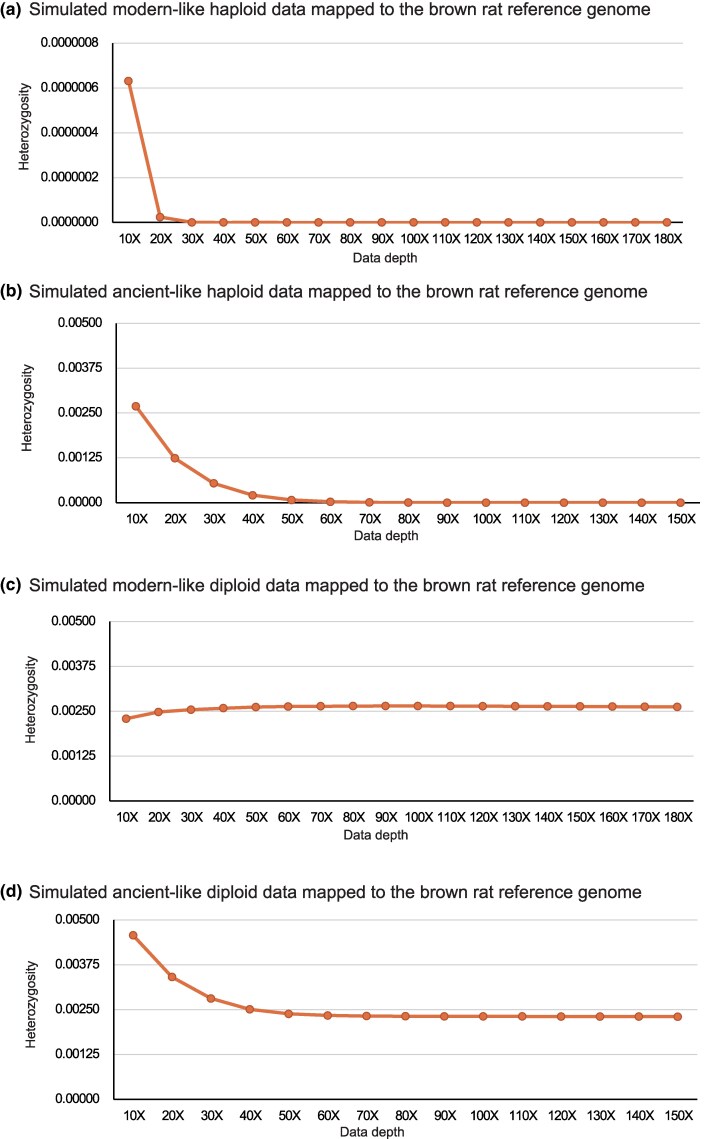
Heterozygosity estimates vary with sequencing depth in same-species mapping. The four panels represent the estimated results using simulated modern-like haploid a), ancient-like haploid b), modern-like diploid c) and ancient-like diploid d) brown rat data. The reference genome is the brown rat reference genome mRatBN7.2.

We first used a high-quality brown rat genome (mRatBN7.2) to simulate both modern- (read length of 100 bp, no misincorporations) and ancient-like (parameterized for endogenous read length and miscoding lesion distribution based on previously published ancient Christmas Island rat data) haploid short-read data (Lin et al. 2022). This was done to investigate the relationship between data quality and sequencing depth at much higher levels than is typically generated from ancient samples. Simulated reads were aligned back to the same brown rat reference genome (Table S1a and S1b, Fig. 1a and 1b), thus representing a haploid, idealized scenario. When sequencing depth reached 50× (modern-like haploid data) and 70× (ancient-like haploid data), genome coverage exceeded 99.19% and plateaued thereafter (Table S1a and S1b). This demonstrates how even when comparing two samples that exhibit no evolutionary divergence, it is not possible to recover a genome at 100% completeness, presumably as a result of the challenge of mapping to complex genome regions and/or aligner algorithmic challenges. As expected, SNP calls between the simulated reads and reference genome were virtually non-existent in the modern-like haploid sequence dataset at high coverage. While low-depth (10×) modern-like haploid data introduced 1,671 spurious SNPs, these false positives were nearly eliminated (only 1 SNP) at 30× depth (Fig. 1a, Table S1a).

For the simulated ancient-like haploid DNA dataset that incorporated damage profiles representative of degraded DNA, false positive SNPs initially remained high, but decreased significantly with increased depth. At ≥110× depth, false positive SNPs fell below 100, a negligible number (Fig. 1b; Table S1b). These findings affirm that high-depth sequencing mitigates damage-induced errors, something especially critical for poor-quality aDNA samples (Briggs et al. 2007; Schubert et al. 2014).

To contextualize this, it is relatively rare that paleogenomic studies sequence specimens to >30×, and even 10× is unusual (Garlapati et al. 2019; Dehasque et al. 2024). In our simulation, even 30×-depth data modeled on damage levels seen in a ca 120 year old historic Christmas Island rat sample, generated over 1.4 million false SNPs (Fig. 1b; Table S1b). This underscores the challenge posed by poorly preserved samples, and the need for stringent SNP filtering criteria in low-depth studies.

To simulate the situation where there is no species-level differentiation between the sequencing reads of the heterozygote and the reference genome, we next repeated our simulations using sequence data from two additional brown rat reference genomes (UTH_Rnor_SHR_Utx and UTH_Rnor_WKY_Bbb_1.0) that were combined at an equal (1:1) ratio, to generate simulated diploid data sets. Both modern- and ancient-like data sets were simulated and aligned to the brown rat reference genome mRatBN7.2 (Table S1c and S1d, Fig. 1c and 1d). As expected, coverage was slightly lower than for haploid models due to sequence divergence between the diploid genome used for simulation, and the reference genome. At 150× simulated sequencing depth, coverage reached 98.56% (modern-like diploid data) and 98.86% (ancient-like diploid data), demonstrating saturation. Notably, SNP frequencies followed distinct trends: in ancient-like diploid simulations, SNP rates declined from 0.4574% at 10×, to 0.2304% at 150×, reflecting the elimination of false positives. In contrast, modern-like diploid samples showed an initial increase in SNPs from 0.2292% (10×) to 0.2646% (90×), followed by a slight decline to 0.2622% at 180× (Fig. 1c and 1d, Table S1c and S1d). This likely reflects the compound effects of increasing detection of true variants alongside a gradual reduction in sequencing-induced errors.

### Cross-Species Mapping

**Fig. 2.**
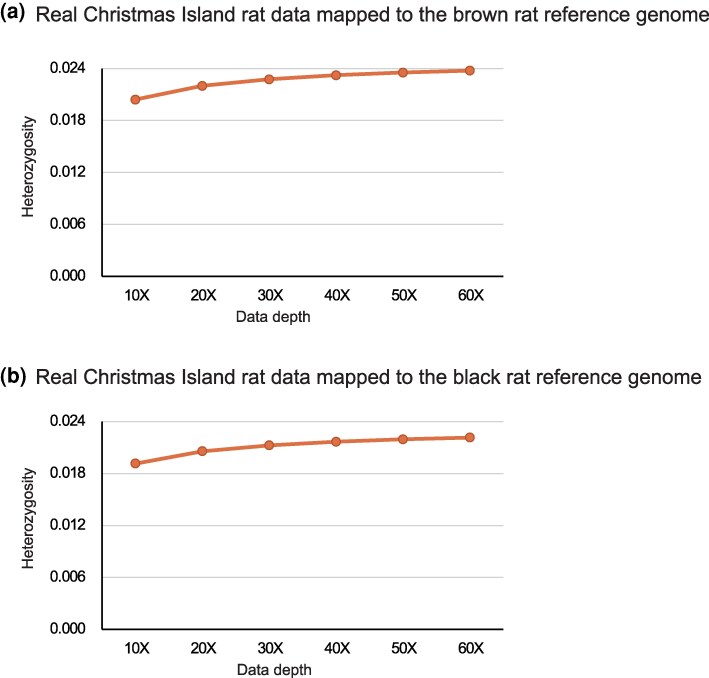
Heterozygosity estimates vary with sequencing depth in Christmas Island rat data mapping results. The panels represent the estimated results of mapping Christmas Island rat data to the brown rat a) and black rat b) reference genome.

In actual de-extinction scenarios, genome editing is unlikely to be used to modify one individual into another from the same species, except for the specific cases involving subspecies or other genomic strain restoration. Instead, most de-extinction efforts will likely be focusing on editing the genome of a closely related extant species to approximate that of an extinct one. This requires the mapping of the extinct species to its closest living relative. Given the evolutionary divergence between the two species, this step introduces losses of key information (Shapiro and Hofreiter 2014; Lin et al. 2022). While we previously demonstrated this effect by mapping sequencing data from the extinct Christmas Island rat to its extant brown rat relative (Lin et al. 2022), we repeated this process by mapping both to the brown rat genome, and that of another close relative, the black rat (*R. rattus*). Consistent with our prior results (Lin et al. 2022), mapping indicated that the maximum average coverage that could be generated with this data is 63.10× for the brown rat (Fig. 2a and Table S2a), while mapping to the black rat genome yielded the higher value of 68.10× (Fig. 2b and Table S2b).

**Fig. 3.**
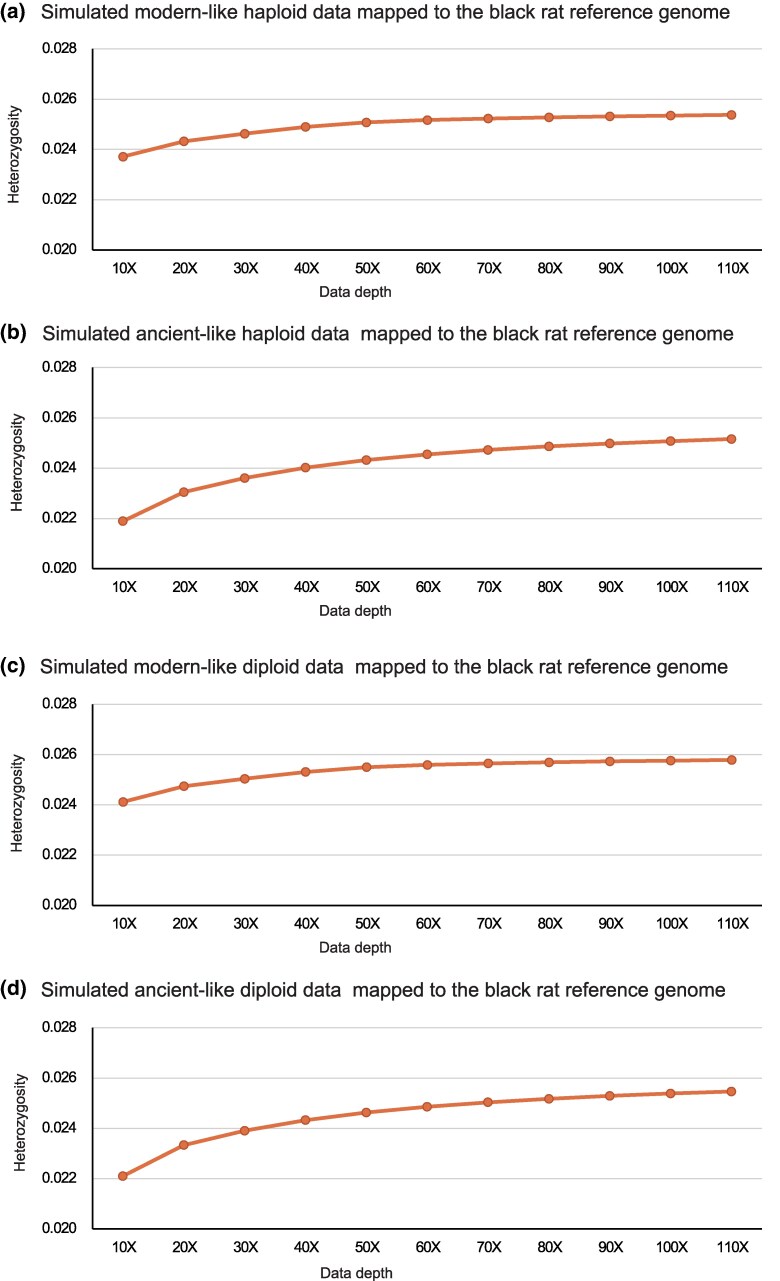
Heterozygosity estimates vary with sequencing depth in cross-species mapping. The four panels represent the estimated results using simulated modern-like haploid a), ancient-like haploid b), modern-like diploid c) and ancient-like diploid d) brown rat data. The reference genome is the black rat reference genome Rrattus_CSIRO_v1.

To explore the limits of using gene editing of close relatives of the Christmas Island rat in order to reconstruct its genome, we then used this data to assess how genome coverage varies with sequencing depth. Using the black rat genome as the reference, we found that even at 10× depth, 93.11% of the black rat genome was covered by at least one read from the Christmas Island rat. However, only 76.72% was covered at ≥5× (suitable for homozygous SNP identification), and just 20.28% at ≥15× (a level typically argued as required for confident heterozygous SNP calls) (Meynert et al. 2014). At 60× depth, these values improved to 96.46% (≥5×) and 88.26% (≥15×), respectively (Table S2b), reiterating the well-accepted fact that high sequencing depth is important for robust variant detection. Overall, this resulted in the identification of 58,979,874 SNPs (2.23% of the genome) and 4,992,996 indels that differ between the Christmas Island and black rat genomes, while comparisons with the brown rat yielded even higher variation: 63,090,834 SNPs (2.38%) and 5,055,673 indels (Fig. 2; Table S2). Although not explicitly examined in this study, in light of prior research, it is highly likely that many of these variants affect coding and regulatory elements (Lin et al. 2022), and thus would influence phenotype (Fig. 3).

Nevertheless, despite these results, we noted that even when the full dataset was used, only 95.35% (brown rat) and 96.56% (black rat) of the respective genomes were covered (Table S2). This implies that even in the best-case scenario (mapping to the black rat), 3.44% of the genome remains unrecoverable. Hence any functional elements or regulatory variants located within these unrecoverable regions would be inherently lost in a hypothetical de-extinction attempt using genome editing based on the black rat genome. This limitation stems from both evolutionary divergence after the last shared common ancestor of the Christmas Island rat and brown rat ∼2.3 million years ago, and technical challenges such as PCR amplification and sequencing biases of AT-rich genomic regions (Aird et al. 2011; Lin et al. 2022). Evolutionary divergence can lead to the insertion or deletion of some genomic fragments, or, due to excessive sequence divergence, cause alignment algorithms to fail in mapping the sequenced reads from an extinct species to the corresponding locations in the reference genomes of extant species.

Given the limited availability of true sequence data, a natural question to ask is whether increasing the sequence depth further would ameliorate the issues? Therefore, we returned to the simulated haploid and diploid datasets representing modern and ancient DNA from the brown rat, and mapped the resulting reads to the black rat reference genome. Unsurprisingly, across all four datasets, coverage and heterozygosity increased with sequencing depth in a consistent manner (Fig. 3, Table S3). Nevertheless, even when sequencing depth reached 110×, only approximately 96% of the black rat genome was covered by at least one read, and only approximately 95% was covered at ≥5× depth (Table S3). This reiterates how mapping challenges introduced by evolutionary divergence cannot be resolved through sequence depth alone.

Consistent with what might be expected, our analysis also revealed that for any sequencing depth, simulated ancient-like diploid data that includes miscoding lesions always exhibited the highest levels of heterozygosity, while simulated modern-like haploid data showed the lowest. Notably however, in all datasets we found that heterozygosity did not reach saturation, even if sequencing depth continued to increase (Fig. 3, Table S3). For example, an increase from 100× to 110× led to a measurable increase of 0.11% to 0.32% in heterozygosity across the different datasets (Table S3). This finding underscores how higher sequencing depth continues to improve variant detection, particularly for ancient samples.

**Fig. 4.**
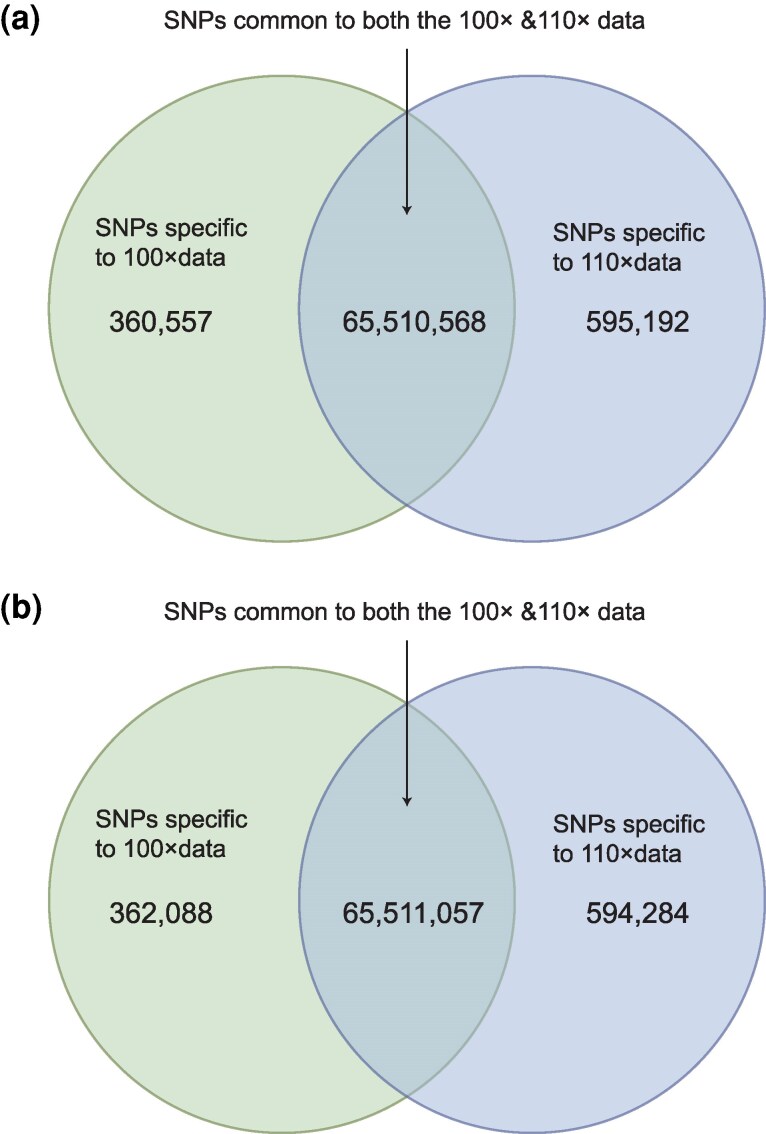
Comparing the SNP profiles from ancient-like diploid datasets simulated at 100× and 110× depth. (a and b) Results obtained from sub-datasets generated by randomly from the simulated ancient-like diploid datasets.

Naturally the distinct behaviors observed for the various datasets reflect differences in both the accumulation of true variants, and the prevalence of false positives. Hence ultimately establishing optimal sequencing depth standards will have to account for sample origin, DNA degradation, and sequencing error profiles. To further understand variant detection at high depths in our data, we compared the SNP profiles obtained after mapping to the reference genome simulated ancient-like diploid datasets generated at 100× and 110× coverage. Surprisingly, we found that while 495,701 new SNPs were detected at 110×, 300,915 SNPs that had been identified at 100× were no longer present, which represents the non-negligible amount of 0.4975% of the total detected SNPs (Fig. 4a). This pattern indicates simultaneous correction of both false positives and false negatives as sequencing depth increased. We then repeated this analysis by generating independent subsampled datasets at 100× and 110×. Again, the results revealed that there was a considerable body of unique sets of SNPs specific to each sequencing depth (Fig. 4b). Although these depth-specific SNPs represent only a small fraction of the genome, they intersect the exonic regions of 3,137 out of 3,256 annotated genes, and some of these genes are associated with critical biological functions and signaling pathways (Tables S3 & S4). It is clear therefore that the challenges associated with mapping aDNA to the genome of closely related species include not only accounting for DNA fragmentation, miscoding lesions, and reduced template complexity (all of which exacerbate alignment errors and compromise variant accuracy), but also relate to the fidelity of cross-species alignment (Shapiro and Hofreiter 2014) and the sequencing depth applied as shown here. The results from our simulation emphasize how high sequencing depth is essential for accurate genome recovery when using a reference genome from a different species.

### Sources of False-Positive SNPs

**Fig. 5.**
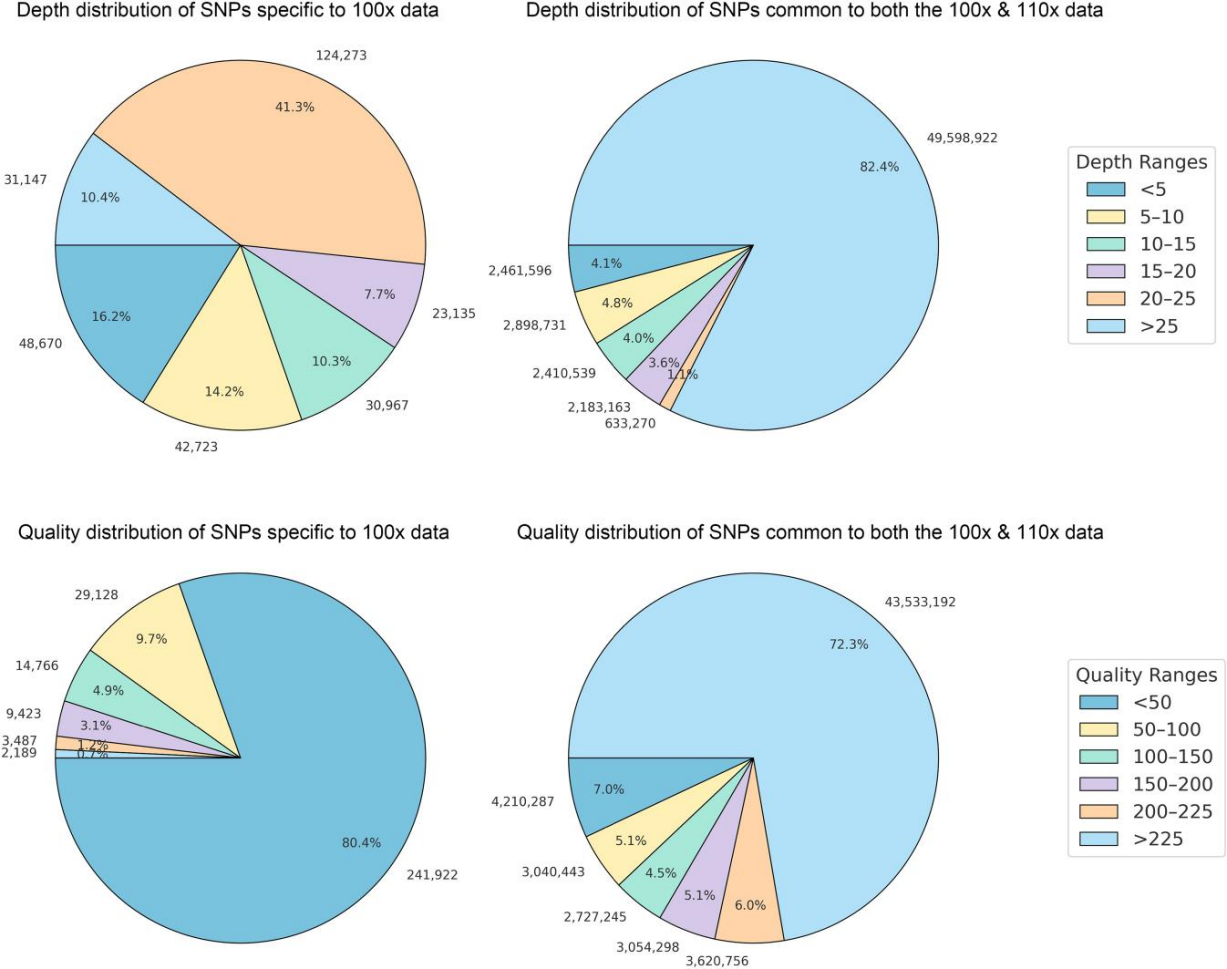
Comparison of sequencing depth and quality scores for SNPs specific to the 100× depth simulated dataset, and SNPs common to both the 100× and 110× depth simulated datasets.

**Fig. 6.**
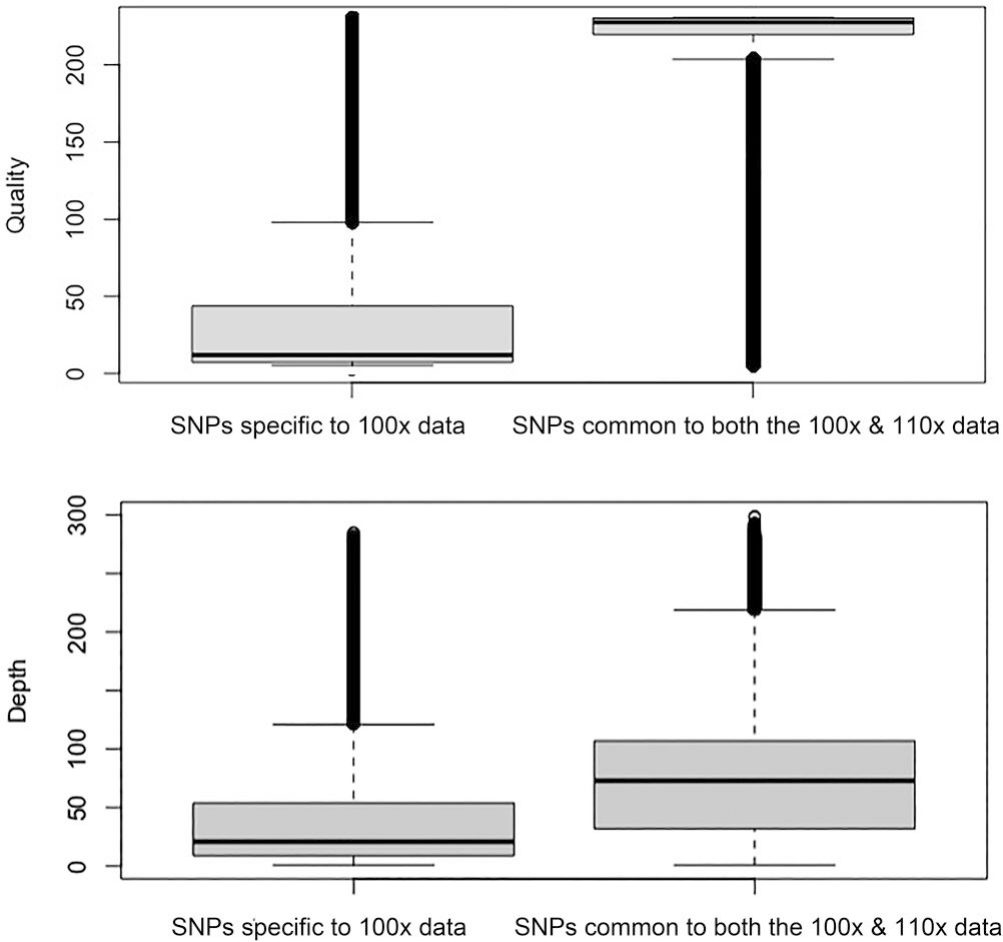
Statistical validation of sequencing depth and variant quality for the SNPs specific to the 100× simulated dataset, and common to both the 110× and 100× depth simulated datasets.

We lastly chose to explore the underlying causes of false-positive single nucleotide polymorphisms (SNPs) in ancient DNA analyses. We simulated haploid and diploid sequencing datasets that had fragment length distributions that were characteristic of ancient DNA, but without the inclusion of damage-derived miscoding lesions (e.g. those derived from cytosine deamination) (Table S6, Fig. S1). Contrary to our expectations, comparing the SNP profiles from these simulated “fragmented but damage-free” diploid datasets at 100× and 110× coverage to black rat reference genome reveals that there are still 351,294 false-positive SNPs at 100× (Fig. S2). This number was comparable to what was observed in the simulated ancient-like DNA datasets that included miscoding lesions (Fig. 4), indicating that DNA fragmentation alone can contribute substantially to erroneous variant calls. We therefore evaluated the quality and depth of SNPs unique to the 100× ancient-like diploid simulated dataset (i.e. not observed in the 110× dataset), versus those shared between the 100× and 110× datasets. The results showed stark differences between these two SNP categories: Specifically, 77.96% of the SNPs specific to 100× data have low quality (Quality < 50), and 86.15% of them are with low depth (Depth < 25). In contrast, 77.32% of the SNPs common to both the 100× & 110× data have high quality (Quality > 200), and 82.23% of them are with high depth (Depth > 25) (Fig. 5). We also compared the depth and the quality, and found that the depth and the quality of the SNPs specific to the 100× data (false positive sites) were significantly (*P* < 2.2e−16) lower than those common to both the 100× and 110× data (Fig. 6). These results indicate that the false positive SNPs were not due to the damage of ancient DNA, but were at least partly caused by low coverage regions.

Our findings suggest that when the sequencing depth reaches a certain level, the primary cause of false-positive SNPs in ancient DNA reconstructions is not damage-related miscoding lesions per se, but rather the presence of low-coverage and low-quality regions, where sequencing uncertainty is the highest. From a methodological perspective, these findings underscore the pivotal role of stringent quality control measures in ancient DNA sequencing data. Despite high overall sequencing depth, local fluctuations in coverage and read quality can introduce systematic biases in variant detection. Therefore, in the context of ancient DNA-based variant calling and genome reconstruction, it is imperative to establish robust strategies for the identification and filtering of low-coverage regions, with more conservative thresholds applied to loci at elevated risk of error. In many ancient DNA studies, bioinformatic pipelines commonly include filters for unmappable regions and extreme depth values; however, the masked regions remain a blind spot in the reconstruction of the genome. Taken together, the results indicate that the occurrence of false-positive SNPs is closely associated with sequencing depth, coverage uniformity, and locus-specific data quality, rather than being solely attributable to damage intrinsic to ancient DNA. The development of a more rigorous variant calling framework, coupled with refined approaches to managing low-quality regions, will be essential for enhancing the accuracy and reliability of genomic reconstructions—particularly in functionally significant regions that inform downstream biological interpretation and decision-making.

## Conclusion

This study used the extinct Christmas Island rat as a model system to evaluate the feasibility, limitations, and sources of error in genome reconstruction using current technologies. By aligning its aDNA sequences to the genomes of closely related species, the black and brown rats, we achieved relatively high mapping depths (63.10× and 68.10×) and genome coverages of 95.35% and 96.56%, respectively (Table S2). Nonetheless, for the black rat reference genome, approximately 3.4% of the genome remained unrecoverable, and over 58 million SNPs and nearly 5 million indels were detected (Table S2, Fig. 2), underscoring the constraints imposed by evolutionary divergence even among closely related taxa.

Simulations across a range of sequencing depths revealed that increasing depth to ≥40× dramatically improved coverage of genomic regions at ≥20× depth, reaching over 99% and enhancing variant detection accuracy. Importantly, further analysis showed that false-positive SNPs arose primarily from low-coverage regions, rather than DNA damage or fragmentation, providing actionable insights into error control in aDNA studies.

Although cross-species mapping can be informative, the effectiveness of this strategy is constrained by evolutionary divergence, DNA damage, and sequencing artifacts. Hence when using paleogenome data in the context of both de-extinction efforts, or more conventional evolutionary genomic analyses, careful optimization of sequencing parameters, and rigorous evaluation of variant detection thresholds are indispensable for successful genome reconstruction. This is relevant because, while it is unlikely that genome-editing based de-extinction projects will reconstruct the exact genome of now-lost species, even attempts to edit small fractions of the genome (e.g. as currently attempted in the Dire Wolf project) (BIOSCIE AL 2025) require highly accurate sequence information. Hence the presence of widely distributed mapping gaps and false positive SNPs that systematically affect a broad range of functional genes complicate such matters. And furthermore, if one turns to the more conventional evolutionary and comparative genomic analyses that are typical of paleogenomic studies of extinct species, then the accuracy of comparisons that can be made are similarly affected by such issues. Therefore, this study provides a theoretical framework that goes beyond de-extinction.

## Materials and Methods

### Data Collection

Three different brown rat (*R. norvegicus*) reference genomes were download from NCBI with accessions GCA_015227675.2 (mRatBN7.2), GCA_023515785.2 (UTH_Rnor_SHR_Utx) and GCA_023515805.2 (UTH_Rnor_WKY_Bbb_1.0). The mRatBN7.2 was used as the reference genome and to generate the haploid sequencing datasets. The other two genomes were used to generate the diploid sequencing datasets. The black rat (*R. rattus*) reference genome (Rrattus_CSIRO_v1) was download from NCBI with accession GCF_011064425.1. Christmas Island rat (*R. macleari*) sequence data was obtained from our previous study (EBI sample accessions SAMEA12813846 and SAMEA12813847).

### Sequence Simulation

The DNA sequence simulator, gargammel, was used to generate simulative modern and simulative ancient brown rat data (Renaud et al. 2017). The simulated reads were set to be single end and 100 bp in length (consistent with the BGISEQ-500 data that had been generated for the Christmas Island rat). For simulated modern data, a fixed fragment length (-l) was set to 100 bp. For simulated ancient data, the size frequency file (*f*) and the miscorporation file (-mapdamage) input values were taken from the estimates made by mapDamage v2.2.1 on the BGISEQ-500 Christmas Island rat data (Jonsson et al. 2013). We first generate each original dataset with a raw data depth of 180× in silico. Then, we created subsets of these data at various sequencing depths through down-sampling to simulate different levels of sequencing depth. The simulated haploid data were generated using mRatBN7.2, which was also chosen to be the reference genome assembly for mapping. In order to approach reality more closely, we utilized two additional reference genomes (UTH_Rnor_SHR_Utx and UTH_Rnor_WKY_Bbb_1.0) of Norwegian brown rats from NCBI, to simulate diploid brown rat (UTH_Rnor_SHR_Utx × UTH_Rnor_WKY_Bbb_1.0) sequencing data which were then aligned to the reference genome (mRatBN7.2). The simulated genome of a diploid individual is derived from two haploid genomes, with each contributing equally (50%).

### Mapping and Calculation of Coverage/Depth

Before mapping the Christmas Island rat data, the last 10 bases of each read from the BGISEQ-500 sequencing perform were removed because they represent the index. Subsequently, the sequence data were trimmed and mapped against the reference genomes of the brown rat (mRatBN7.2) and black rat, using Paleomix v1.3.2 (Schubert et al. 2014). Specifically, the adapters in BGISEQ-500 data (adapter1: AAGTCGGAGGCCAAGCGGTCTTAGGAAGACAA; adapter2: GAACGACATGGCTACGATCCGACTT) and Illumina data (adapter1: AGATCGGAAGAGCACACGTCTGAACTCCAGTCACNNNNNNATCTCGTATGCCGTCTTCTGCTTG; adapter2: AGATCGGAAGAGCGTCGTGTAGGGAAAGAGTGTAGATCTCGGTGGTCGCCGTATCATT) were trimmed by AdapterRemoval v2.3.1 with default setting (Schubert et al. 2016). bwa v0.7.17 (the backtrack algorithm) was used to map the reads to the genome with options “MinQuality: 0; Filter UnmappedReads: yes; Used Seed: no” (Li and Durbin 2009). In the mapping step, while we used the same settings for modern and ancient samples a different aligner algorithm was used. Specifically, BWA-men was used for modern data, and BWA-backtrack for ancient data to ensure a good alignment effect. For the ancient samples, mapDamage (Jonsson et al. 2013) was used to estimate the ancient DNA damage parameters, to both validate that the data is truly ancient and to provide input values for the gargammel simulations. In our previous research, we recovered the mtDNA consensus sequences from each of the Christmas Island specimens, and compared them using MEGA X (Kumar et al. 2018) and found that they exhibited very little genetic distance (0.002890), thus we elected to merge the two sequence datasets to obtain the final high coverage data set (Lin et al. 2022) . The bam files generated from each species were merged into one bam file using samtools v1.9 (Li et al. 2009). We used paleomix coverage and Paleomix depths to calculate the coverage and depth histogram for a bam file. The “bedtools coverage” command in bedtools v2.29.0 was used to calculate the coverage rate of each gene in the brown rat genome (Quinlan and Hall 2010). Allele frequency spectrum estimation was performed using ANGSD (with minimum mapping quality 30, minimum base quality 20, followed by realSFS optimization, while SNP and indel variant calling was conducted using bcftools; Danecek et al. 2021).

## Supplemental Material

Supplementary material is available at *Genome Biology and Evolution* online.

## Supplementary Material

evaf251_Supplementary_Data

## Data Availability

Three different brown rat (*Rattus norvegicus*) reference genomes were downloaded from NCBI with accessions GCA_015227675.2 (mRatBN7.2), GCA_023515785.2 (UTH_Rnor_SHR_Utx) and GCA_023515805.2 (UTH_Rnor_WKY_Bbb_1.0). The black rat (*Rattus rattus*) reference genomes were downloaded from NCBI with accession GCF_011064425.1. Christmas Island rat (*Rattus macleari*) sequence data was obtained from our previous study (EBI sample accessions SAMEA12813846 and SAMEA12813847). The source code related to this manuscript has been deposited and is publicly available in the GitHub repository at: https://github.com/LinJianQing-ST/De-Extinction_Genomics_Code.
