# Supplementary material for: Mapping the Genomic Limits of De-Extinction in the Face of Ancient DNA Degradation
Source: Genome Biol Evol. 2026 Jan 3;18(1):evaf251. doi: 10.1093/gbe/evaf251 (PMC12794020; doi:10.1093/gbe/evaf251)
Supplement: evaf251_Supplementary_Data [file evaf251_supplementary_data.zip › Supplemental Figures.docx]

**Supplemental Figures**


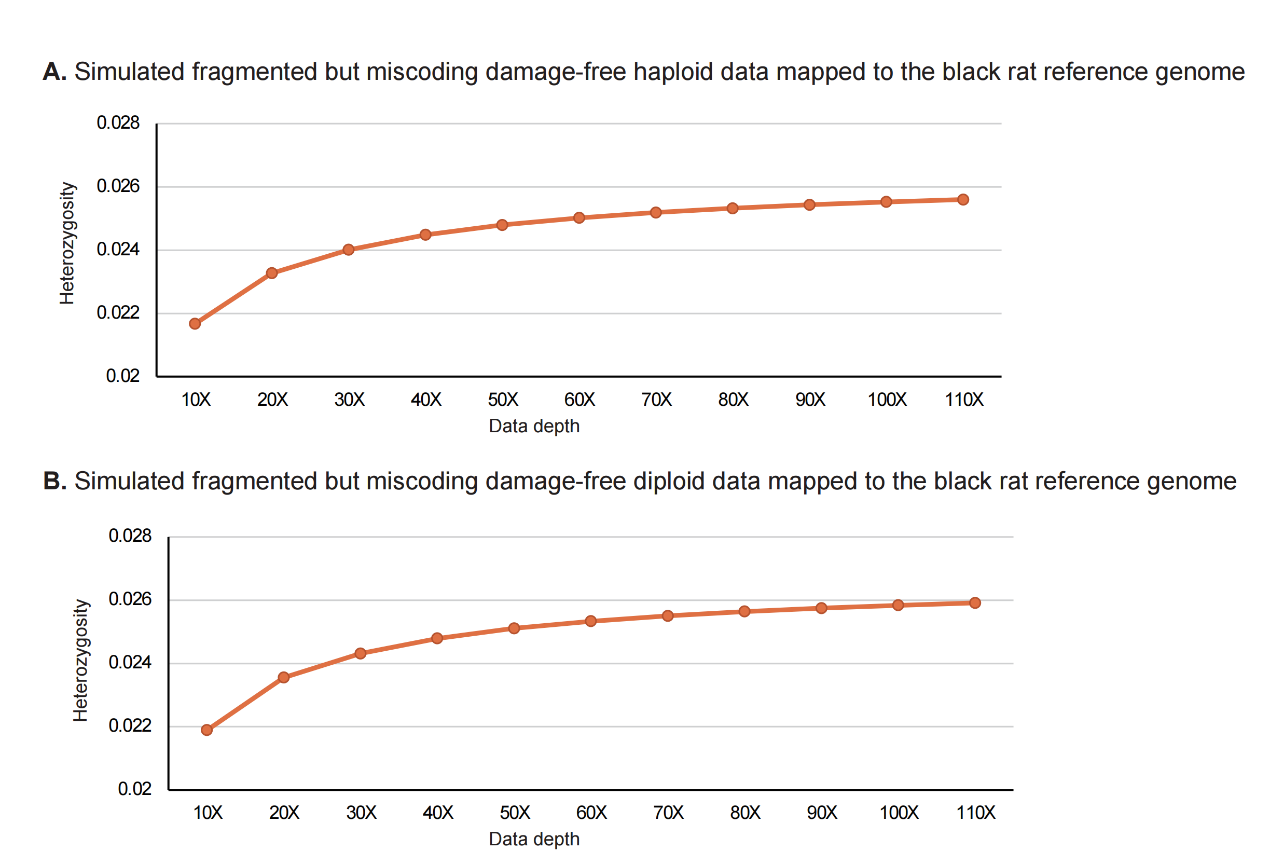


**Figure S1 Results when mapping fragmented, miscoding damage-free, DNA to the black rat reference genome.**


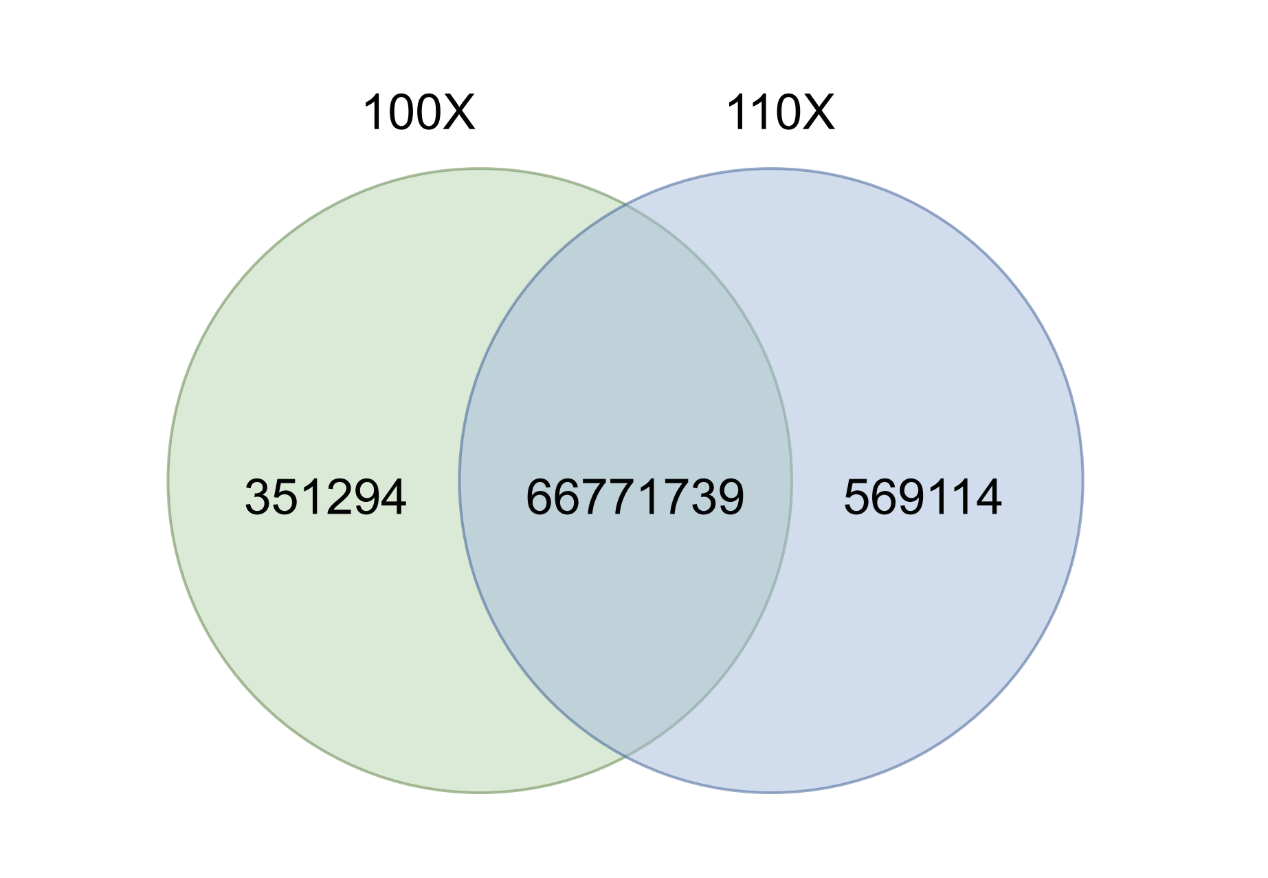


**Figure S2 Comparing the SNP profiles from fragmented, miscoding damage-free DNA data simulated at 100× and 110× depth.**
